# A Case of Poisoning with Oil of Tansy

**Published:** 1852-08

**Authors:** 


*A Case of Poisoning with Oil of Tansy—Death at the end of three hours and a-half—Quantity of the Drug taken about ℥j and ʒiii.* By John C. Dalton, Jr., M. D. (Read to the Boston Society for Medical Observation, June 2d, 1851.)

E. S., a fine, healthy-looking girl, about twenty-one years of age, died at the house of Mr. A., in Boston, on Wednesday, the 7th of May, 1851. She had been employed in Mr. A.'s family as a seamstress since some time in the previous winter, living in the house during the week, but going away on Saturdays to a cousin's in Pleasant street, and returning to Mr. A.'s on Monday morning. She had been for some months receiving the attentions of a young man who was reputed to be engaged to her. None of her friends, however, suspected anything to be wrong with her until Monday, May 5, when her cousin, with whom she had been spending Sunday as usual, perceived the odor of tansy in the room which she had occupied; whereupon it occurred to her that the girl might have become pregnant, and used the drug for the purpose of producing abortion.

On Tuesday, she was engaged in her ordinary employment, and dined heartily a little after five o'clock in the afternoon. She went up stairs to her room about half-past nine o'clock. The cook, who occupied a room above, went up with her and stopped in her room, conversing, for some fifteen minutes. The girl's manner was perfectly natural and cheerful, as it had been throughout the day. About a quarter before ten o'clock the cook left her preparing for bed, and went up to her own room.

Nothing more was heard from her till about eleven, when Mr. and Mrs. A., who were sitting in the basement-room, heard a scream, which they supposed to come from one of the children. Mrs. A. went immediately up stairs, and on entering Miss S.'s room found her on the floor, by the side of the bed, insensible and in violent convulsions. She had evidently fallen out of bed, as she was undressed, and the bedclothes were disturbed, and had been partially dragged on to the floor with her. Dr. Morrill was immediately sent for, and arrived in about ten minutes. He sent also for me, and I arrived at the house at half-past eleven o'clock.

The girl was then lying on her back by the side of the bed, and presented the following appearances: Total unconsciousness; cheeks flushed, of a bright red color; eyes open and very brilliant; pupils of equal size, widely dilated and immovable; sclerotics injected; skin warm, not remarkable as to moisture; respiration hurried, labored, stertorous, and obstructed by an abundance of frothy mucus, which filled the air-passages, and was blown from between the lips in expiration; the breath had a strong odour of tansy, as had been already observed by Dr. Morrill; pulse quite full, forcible, 128; at intervals of five to ten minutes the body was convulsed by strong spasms, in which the head was thrown back, the respiration suspended, the arms raised and kept rigidly extended, and the fingers contracted. After this state of rigidity had continued for about half a minute, it was usually succeeded by a tremulous motion, often sufficient to shake the room, together with very faint and imperfect attempts at inspiration. The whole interval from the commencement of the convulsion to the first full inspiration, varied from a minute to a minute and a-half. Occasionally, the tongue was wounded by the teeth, and the saliva slightly tinged with blood. Immediately after a convulsion the countenance was very pallid and livid, from the suspension of respiration, and the pulse exceedingly reduced in strength and frequency. The pulse and color then gradually returned until the occurrence of the next spasm. It was very common, a few seconds after the termination of a convulsion, for the head to be drawn slowly backward, and the eyelids, at the same time, stretched wide open. In the intervals of the convulsions, the limbs were mostly relaxed, but the jaws remained clenched.

A vein was immediately opened in the right arm, and about Oij of blood taken away. After this, the pulse became much softer and the face lost its bright color. There was, however, no change in the condition of the pupils, nor return of consciousness, nor other improvement in the appearance of the patient. It being impossible to get anything down the throat, two injections of an ounce of wine of antimony, with about ʒss of powdered ipecac., were thrown up the rectum at intervals of about half an hour, but produced no apparent effect.

On searching the room, a ℥ij phial was found in the pocket of the girl's dress, wrapped in a piece of paper, labelled "Oil of Tansy," and marked with the name and address of an apothecary in Pleasant street. The phial contained ʒv of oil of tansy of the ordinary purity. A mug was also found, from which she had apparently drunk the oil mixed with water, as it smelt very strongly of the drug, and still had a drop or two of it at the bottom.

The condition of the patient continued much the same for about an hour. The convulsions, however, gradually became less protracted, and the failure of the pulse after each attack, more complete, at the same time that it recovered strength less perfectly in the intervals. The countenance also became somewhat sunken and the temperature of the skin reduced. About 1 o'clock, six leeches were applied to the forehead and temples, and sinapisms put on the calves of the legs. The leech-bites bled freely.

Toward 2 o'clock the alteration for the worse became quite rapid. Pulse 124 and feeble; respiration 36, and attended with less muscular effort than at first; the left cornea was glazed, but the right continued brilliant; a little inward strabismus of the right eye, and the mouth and nose drawn a little to the right side. Occasionally, a slow, lateral, rolling motion of the eyeballs. At five minutes past two she had the last convulsion, which was much less violent than the earlier ones, and lasted only half a minute. There was no recovery of the pulse after this attack, and she died at a quarter-past two o'clock, A. M.

*Autopsy* ten hours after death.

Countenance natural; cadaveric rigidity very strong; only slight purplish discoloration of dependent parts; no ecchymoses anywhere; no effect had been produced by the sinapisms on the legs.

*Head.*—Scalp not injected; distinct, but not excessive dryness of arachnoid over hemispheres of brain; no effusion, congestion or other unnatural appearances anywhere about encephalon.

*Chest.*—Heart and pericardium natural; left ventricle firmly contracted; blood everywhere unusually fluid; interior of heart exhaled a distinct odor of tansy, as also cut surface of pectoral muscles.

No alteration of pleura; lungs rather shrunken, crepitated perfectly everywhere, and were not at all engorged; air-passages not remarkable except for a little redness of posterior surface of epiglottis.

*Abdomen.*—Strong odor of tansy in peritoneal cavity; a few drachms of thin fluid in pelvis; peritoneum natural in appearance.

Œsophagus natural internally, except that epithelium was somewhat deficient in lower part.

The stomach contained about ℥xij of a semifluid, yellowish-gray substance, consisting of partially digested food, potato, cranberries, onions, &c., mixed with an abundance of small brownish-yellow, glistening oil globules, and exhaling an excessive odor of tansy; mucous membrane generally pale, not vascular in any part, but throughout nearly the whole of great pouch brownish and much thinned and softened, so that for a considerable space it is nearly or quite destroyed. There was an old, whitish, slightly puckered cicatrix of the mucous membrane on posterior wall of stomach, near small curvature. No other morbid appearance.

The lacteals of the mesentery were very distinct, and distended with milky chyle.

Small intestines were natural internally throughout. They contained, at their upper part, pasty masses of dusky-colored chyme, mixed with oil of tansy.

Below, the contents were less abundant, and were unmixed with oil.

Large intestine contained yellowish feces, and small masses of a brownish powder, apparently ipecac. Mucous membrane natural.

Spleen rather shrunken, flabby, and deficient in blood. Other abdominal organs not remarkable except for slight paleness.

Urinary bladder contained ℥ii to ℥iii of urine.

The uterus was enlarged, so that its upper edge came two and three-quarter inches above level of symphysis pubis. It contained a well-formed female fœtus, about four months old.

There was not the least appearance anywhere of the fœtus or membranes having suffered any disturbance.

The left ovary, which hung down a little lower than the right, had near its external extremity a small conical prominence, where the fibrous coat was wanting, and its place occupied by peritoneum alone. There was a very slight appearance here of a cicatrix, visible only on close inspection. There was no unusual vascularity here, or at any other part of the ovary. Beneath this prominence the corpus luteum could be felt through the ovarian tissue, tolerably firm and well defined, and having the form of a sphere, compressed laterally, much like that of the crystalline lens. On dividing the ovary longitudinally through the prominence, the corpus luteum was exposed. It presented a nearly circular section, measuring seven-eighths of an inch in its long diameter, and three-fourths of an inch in its short. It consisted externally of a convoluted wall, of a dull-yellow color, measuring at its deepest part a little over three-sixteenths of an inch in thickness. The space enclosed by the yellow wall was occupied by a colorless, reticulated, fibrinous coagulum, which possessed a few minute vessels. This central coagulum was much compressed laterally; so that, although it presented a cut surface of about half an inch in diameter, it had hardly more than one line in thickness. There was no cavity nor fluid anywhere.

Both ovaries were carefully divided in every direction, but only one other body was found having any resemblance to a corpus luteum, and that was so small and imperfect as to be hardly recognizable. There were many Graafian vesicles in the interior of each ovary, varying in diameter from threesixteenths of an inch downward, but none at all prominent on the surface. Both ovaries were quite healthy.

It was subsequently ascertained that the oil of tansy was obtained, at the shop of the apothecary whose label it bore, on the evening of Friday or Saturday preceding the girl's death. The apothecary's clerk, who recognized the bottle, testified at the inquest that he put up in it ℥ij of oil of tansy, and delivered it to a girl about fourteen years old, who stated that the family that sent for it wished to take it into the country. The patient, therefore, undoubtedly took ℥i and ʒiij of the drug. It seems probable that the violent action of the poison commenced at eleven o'clock, at the time the family heard the patient scream; and if we allow fifteen minutes for the absorption of the oil after it was swallowed, it would give three hours and a-half from the time of taking the drug till the patient's death. Fifteen minutes may seem rather a long time for the operation of a volatile oil to be delayed, but it is probably no more than should be allowed. In a case which recently came under the notice of Dr. Dalton, of Lowell, a girl took a quantity of oil of tansy just before dinner. She then went into the dining-room, sat some time at the table, ate with apparent relish, felt sick, left the table, went into the yard, vomited what she had eaten, and immediately fell down insensible and convulsed. She recovered, after remaining a long time unconscious. The only other recorded fatal case of poisoning with this oil that I am acquainted with also occurred in Boston, under the care of Dr. C. T. Hildreth, and was published in the *American Journal of Med. Sciences* for May 1835. In that case the woman took ℥ss of the drug, and did not lose consciousness entirely till three-quarters of an hour afterward. although she was convulsed at intervals before that time. After unconsciousness became complete, she did not again recover it, and died rather less than two hours after taking the poison.

The present case is another instance of the extreme violence to which the system may be subjected even in the early months of pregnancy, without inducing abortion. Though all the muscles, both of the body and limbs, were for three hours and a quarter subjected to a succession of the most violent contractions, there was no sign of abortion, and after death the ovum was found in the uterus entirely undisturbed. In Dr. Hildreth's case, also, pregnancy existed but a few weeks advanced, and the drug was undoubtedly taken for the purpose of producing abortion, but nothing of the kind took place. The general symptoms in that case were similar to those described in the foregoing, the most remarkable difference being the more gradual loss of consciousness, and the more rapid death after a much smaller dose.—*Am. Jour. Med. Sciences.*

